# Non-hypervascular hepatobiliary phase hypointense lesions detected in patients with hepatocellular carcinoma: a post hoc analysis of SORAMIC trial to identify risk factors for progression

**DOI:** 10.1007/s00330-022-09000-1

**Published:** 2022-07-26

**Authors:** Osman Öcal, Christoph J. Zech, Matthias P. Fabritius, Christian Loewe, Otto van Delden, Vincent Vandecaveye, Bernhard Gebauer, Thomas Berg, Christian Sengel, Irene Bargellini, Roberto Iezzi, Alberto Benito, Maciej Pech, Antonio Gasbarrini, Bruno Sangro, Peter Malfertheiner, Jens Ricke, Max Seidensticker

**Affiliations:** 1 Department of Radiology, University Hospital, Ludwig Maximilian University of Munich, Marchioninistrasse 15, 81377 Munich, Germany; 2 Radiology and Nuclear Medicine, University Hospital Basel, University of Basel, Basel, Switzerland; 3 Section of Cardiovascular and Interventional Radiology, Department of Bioimaging and Image-Guided Therapy, Medical University of Vienna, Vienna, Austria; 4 Department of Radiology and Nuclear Medicine, Academic University Medical Centers, University of Amsterdam, Amsterdam, The Netherlands; 5 Department of Radiology, University Hospitals Leuven, Leuven, Belgium; 6 Department of Radiology, Charité – University Medicine Berlin, Berlin, Germany; 7 Klinik und Poliklinik für Gastroenterologie, Sektion Hepatologie, Universitätsklinikum Leipzig, Leipzig, Germany; 8 Radiology Department, Grenoble University Hospital, La Tronche, France; 9 Department of Vascular and Interventional Radiology, University Hospital of Pisa, Pisa, Italy; 10 Fondazione Policlinico Universitario A. Gemelli IRCCS, UOC di Radiologia, Dipartimento di Diagnostica per Immagini, Radioterapia Oncologica ed Ematologia, Rome, Italy; 11 Abdominal Radiology Unit, Department of Radiology, Clínica Universidad de Navarra, Pamplona, Spain; 12 Departments of Radiology and Nuclear Medicine, University of Magdeburg, Magdeburg, Germany; 13 Fondazione Policlinico Universitario Gemelli IRCCS, Universita’ Cattolica del Sacro Cuore, Rome, Italy; 14 Liver Unit, Clínica Universidad de Navarra and CIBEREHD, Pamplona, Spain; 15 Department of Medicine II, University Hospital, LMU Munich, Munich, Germany

**Keywords:** Magnetic resonance imaging, Gadoxetic acid, Hepatobiliary phase, Hepatocellular carcinoma, Hypovascular hypointense lesions

## Abstract

**Objectives:**

To identify clinical and imaging parameters associated with progression of non-hypervascular hepatobiliary phase hypointense lesions during follow-up in patients who received treatment for hepatocellular carcinoma.

**Methods:**

A total of 67 patients with 106 lesions were identified after screening 538 patients who underwent gadoxetic acid–enhanced MRI within the SORAMIC trial. All patients were allocated to the trial treatment according to the trial scheme, and 61 of 67 patients received systemic treatment with sorafenib (either alone or combined with locoregional therapies) during the trial period. Follow-up images after treatment according to trial scheme were reviewed for subsequent hypervascularization or > 1 cm size increase. The correlation between progression and several imaging and clinical parameters was assessed using univariable and multivariable analyses.

**Results:**

On a median 178 (range, 48–1072) days follow-up period, progression was encountered in 18 (16.9%) lesions in 12 (17.9%) patients. In univariable analysis size > 12.6 mm (*p* = 0.070), ECOG-PS (*p* = 0.025), hypointensity at T1-weighted imaging (*p* = 0.028), hyperintensity at T2-weighted imaging (*p* < 0.001), hyperintensity at DWI images (*p* = 0.007), and cirrhosis (*p* = 0.065) were correlated with progression during follow-up. Hyperintensity at T2 images (*p* = 0.011) was an independent risk factor for progression in multivariable analysis, as well as cirrhosis (*p* = 0.033) and ECOG-PS (*p* = 0.030).

**Conclusions:**

Non-hypervascular hepatobiliary phase hypointense lesions are associated with subsequent progression after treatment in patients with HCC. T2 hyperintensity, diffusion restriction, cirrhosis, and higher ECOG-PS could identify lesions with increased risk. These factors should be considered for further diagnostic evaluation or treatment of such lesions.

**Key Points:**

*• Non-hypervascular hepatobiliary phase hypointense lesions have considerable risk of progression in patients with hepatocellular carcinoma receiving treatment.*

*• T2 hyperintensity, cirrhosis, ECOG-PS, and hyperintensity at DWI are associated with increased risk of progression.*

*• Non-hypervascular hepatobiliary phase hypointense lesions should be considered in the decision-making process of locoregional therapies, especially in the presence of these risk factors.*

**Supplementary Information:**

The online version contains supplementary material available at 10.1007/s00330-022-09000-1.

## Introduction

Non-invasive diagnosis of hepatocellular carcinoma (HCC) is based on arterial hypervascularity and wash-out on the venous phase in CT or MRI. However, while offering a high specificity (up to 100%), diagnosis of HCC by these criteria lacks sensitivity (40–80%) [1–5]. Gadoxetic acid–enhanced MRI offers improved detection of HCC lesions, as well as detection of non-hypervascular precursor lesions by lack of uptake compared to normal hepatic parenchyma [6, 7]. However, current classification systems do not consider lesions detected on hepatobiliary phase imaging lacking arterial enhancement without histological proof [8, 9] mainly due to imperfect specificity which is not accepted in the environment of organ allocation for liver transplant. However, this does not reflect necessities of disease burden assessment in patients planned for locoregional treatments. Several studies have shown that the presence of lesions that are non-hypervascular but hypointense in hepatobiliary phase is associated with an increased risk of recurrence after local ablation or surgical resection [10], and pathological examination of these lesions has shown that up to 44% of these lesions are actually overt HCC and only 8% benign [6]. A systematic review of sixteen publications that evaluated patients with cirrhosis has shown that 28.2% of non-hypervascular hepatobiliary phase hypointense lesions progressed into overt HCC during follow-up [11]. Yet, there is only scarce available data in the literature, and most of the publications comprise Asian cohorts. Thus, more data for better characterization and definition of the exact role of these lesions are needed. Moreover, to the best of our knowledge, there is no data published from a patient cohort under systemic treatment with sorafenib. Our study aimed to identify clinical and imaging risk factors of progression in non-hypervascular hepatobiliary phase hypointense lesions in patients who underwent treatment for HCC within a randomized-controlled trial.

## Materials and methods

Sorafenib and Micro-Therapy Guided by Gadolinium-EOB-DTPA-Enhanced MRI (SORAMIC; EudraCT2009-012576-27; NCT01126645) is a prospective, phase II randomized-controlled trial conducted at 38 centers in 12 countries in Europe that comprises three sub-study arms: diagnostic (comparing gadoxetic acid–enhanced MRI with CT for patient stratification to local ablation or palliative therapies) [12]; local ablation (evaluating the value of adjuvant sorafenib after radiofrequency ablation); and palliative (evaluating the impact of additional radioembolization to sorafenib on overall survival) [13]. The inclusion criteria for SORAMIC were HCC diagnosis confirmed by either histopathological evaluation or non-invasive imaging criteria, Barcelona Clinic Liver Cancer (BCLC) stage A-C, Child-Pugh scores A or B7 liver functions, and an Eastern Cooperative Oncology Group performance status (ECOG-PS) ≤ 2. In the palliative arm, extrahepatic metastases were permitted if the disease was liver dominant and did not involve the lungs.

### Imaging protocol

Within the diagnostic arm of the trial, all patients underwent contrast-enhanced CT and gadoxetic acid–enhanced MRI using a standardized protocol. CT protocol started with pre-contrast (not mandatory) images followed by injection of contrast media with a speed of 4 mL/s (total 100–150 mL) and arterial phase (15 s after reaching 100 HU in the descending aorta), portovenous phase (50-s trigger delay), and venous phase (120 s after the start of injection) images. The gadoxetic acid MRI protocol consisted of pre-contrast T1-weighted gradient echo (GRE) sequences acquired 2D and 3D, which was followed by an injection of 0.1 mL/kg gadoxetic acid with an injection rate of 1.5 mL/s followed by a 30-mL saline flush, and the dynamic series in the late arterial phase (15 s), portovenous phase (60–70 s), and venous phase (120 s). At 20 min after contrast injection, T1-weighted GRE 2D and 3D hepatobiliary phase images were acquired. Between the dynamic series and the hepatobiliary phase, T2-weighted turbo spin-echo 2D sequences and diffusion-weighted imaging (not mandatory) were performed.

A total of 538 patients underwent a blinded image read by two reader groups, and in addition to typical HCC lesions, the presence of hepatobiliary phase hypointense lesions without arterial phase enhancement was recorded, and lesions were marked. In this pre-planned post hoc analysis, patients with hypovascular hepatobiliary phase hypointense lesions detected by any reader groups were further analyzed. The study protocol was approved by the institutional review board of participating centers and competent authorities. Written informed consent was obtained from all patients.

Follow-up imaging every 2 months with both CT and MRI using the same protocol with the pretreatment images was mandatory in the local ablation arm. In the palliative arm, follow-up imaging every 3 months was recommended but was not a mandatory part of the SORAMIC trial. The imaging modality—computed tomography (CT) or magnetic resonance imaging (MRI)—was chosen by the local investigator. However, similar to the local ablation arm, the same standardized imaging protocol was used.

### Study population

**Fig. 1:**
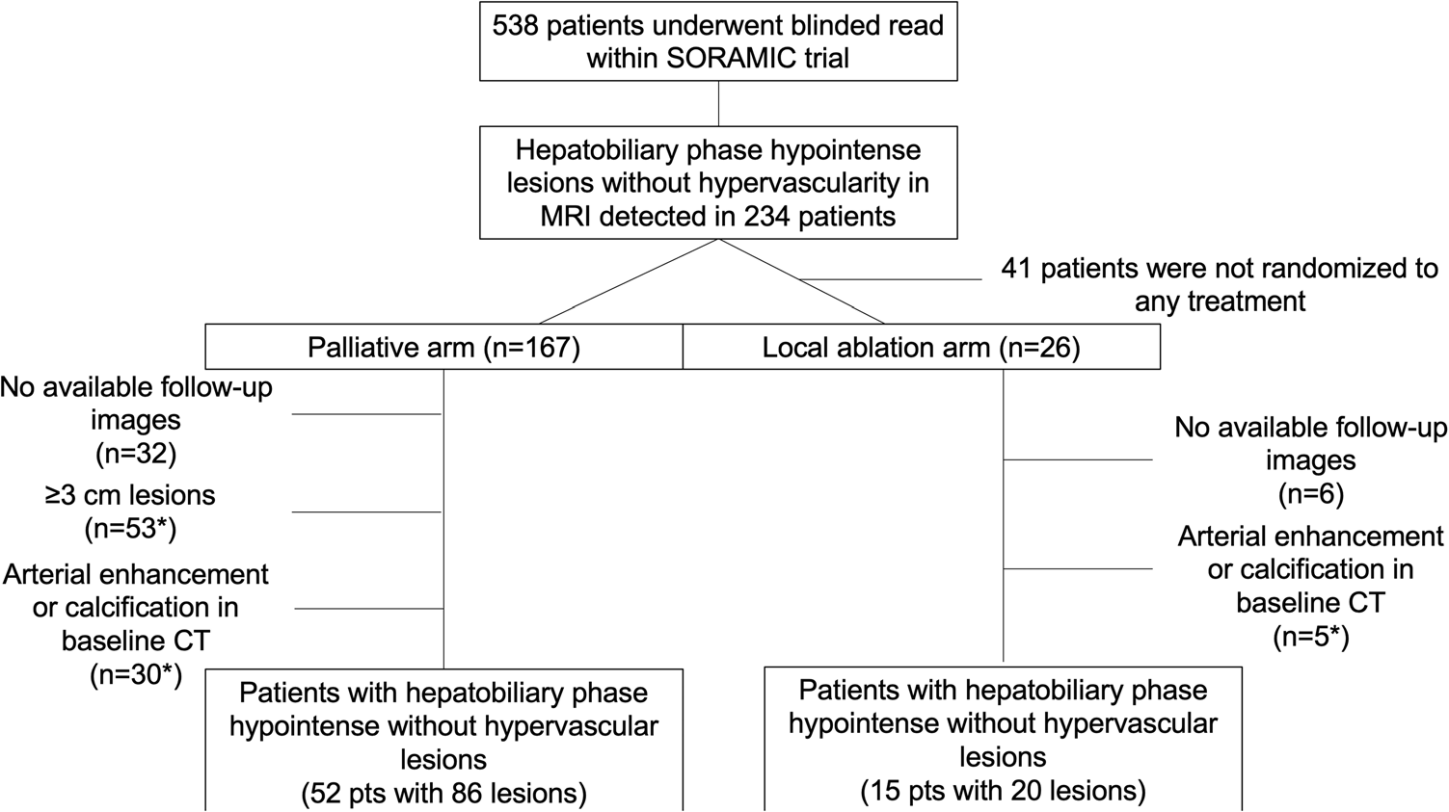
Consort diagram. *Number of patients

In 234 patients, at least one hepatobiliary phase hypointense lesion without hypervascularity was detected. Forty-one patients were not randomized to any treatment arm and excluded from this analysis due to reasons listed in Supplementary Table 1. Pretreatment images of the remaining 193 patients were reviewed by two further radiologists (O.Ö. and M.P.F.), and the lesions marked as hypovascular hepatobiliary phase hypointense were evaluated. Lesions larger than ≥ 3 cm, patients without any follow-up images, and lesions with calcification or arterial enhancement on CT were excluded. In case of discrepancy between two radiologists, images were evaluated by an additional radiologist with > 10 years of experience in abdominal imaging (M.S.). The remaining 67 patients with 106 hypovascular hepatobiliary phase hypointense lesions comprised the final study population (Fig. 1). While 52 patients with 86 lesions were allocated to the palliative arm (sorafenib ± radioembolization), 15 patients with 20 lesions were allocated to the local ablation arm (RFA + sorafenib/placebo). Baseline characteristics of the study population are summarized in Table 1. The mean age was 67.5 (range, 46–84) years, and 60 (89.5%) patients were male. Fifty-two (77.6%) patients had cirrhosis; underlying etiology was hepatitis B in 6 (8.9%), hepatitis C in 11 (16.4%), and alcoholic liver disease in 31 (46.2%) patients. The mean diameter of the hepatobiliary phase hypointense lesions without hypervascularity at the baseline images was 11.5 (range, 4.2–22.5) mm. All lesions were LI-RADS category LR-4 at the baseline images.

**Table 1:** Patient characteristics

Characteristics		*n* = 67
Age (mean, range)		67.5, 46–84
Sex (male)		60 (89.5%)
Cirrhosis		52 (77.6%)
Etiology		
Hepatitis B		6 (8.9%)
Hepatitis C		11 (16.4%)
Alcohol		31 (46.2%)
BCLC		
A		12 (17.9)
B		20 (29.8)
C		35 (52.3)
Child-Pugh class		
A		63 (94.1)
B		4 (5.9)
Treatment		
Local ablation arm	RFA	1 (1.5)
	RFA + placebo	4 (5.9)
	RFA + sorafenib	10 (14.9)
Palliative arm	SIRT	1 (1.5)
	SIRT + sorafenib	21 (31.3)
	Sorafenib	30 (44.8)

### Study treatment

In the palliative arm, patients randomized to sorafenib monotherapy were administered sorafenib with a starting dose of 200 mg b.i.d. for 1 week. The dose was increased to the target dose of 400 mg b.i.d. after 1 week. The sorafenib dose was modified according to pre-defined dosing guidelines in case of drug-related toxicity. In patients randomized to the combination of radioembolization and sorafenib arm, sorafenib treatment was initiated 3 days after the last radioembolization session. In the local ablation arm, all HCC lesions were treated with radiofrequency ablation (RFA). Patients randomized to the experimental arm were started with sorafenib after the last RFA session using the same protocol with the palliative arm. In the control arm, patients received placebo. Sorafenib treatment was continued until disease progression (evaluated by the local investigator) or toxicity which required discontinuation. In summary, from the 67 patients, 61 received systemic treatment with a target dose of 400 mg sorafenib throughout the follow-up period.

### Image analysis

Each hepatobiliary phase hypointense lesion without hypervascularity in these 67 patients was evaluated by two radiologists (O.Ö. and M.P.F.) blinded to all clinical information in pretreatment MRI images for lesion diameter, T1 and T2 signal intensity compared to adjacent liver parenchyma, and presence of intralesional fat as a signal drop on out-of-phase compared to in-phase images. When available, DWI signal intensity was evaluated qualitatively compared to adjacent parenchyma. After this, the same radiologists evaluated all follow-up images, and the development of arterial phase enhancement or more than 1 cm size increase was recorded. In case of discrepancy between the two readers, images were evaluated by an additional reader (M.S.), and the decision was reached by the majority of votes.

### Statistical analysis

All statistical analyses were performed using R statistical and computing software, version 3.5.0 (http://www.r-project.org). Categorical variables were reported as counts and percentages, and continuous variables as means and standard deviations or median and range. Chi-square and Fisher’s exact tests were applied to compare patient characteristics. A receiver operating characteristic (ROC) curve was generated for the initial lesion diameter to predict progression. Significance was set as a two-sided *p* value of < 0.05. Multivariable logistic regression analysis was performed to identify independent predictors of progression among variables with a *p* value of less than 0.1 in the univariable analyses. Interreader agreement of imaging features was calculated by using Cohen simple _*K*_ statistic and was interpreted as follows: 0–0.20, slight agreement; 0.21–0.40, fair agreement; 0.41–0.60, moderate agreement; 0.61– 0.80, substantial agreement; and 0.81–1.00, almost perfect agreement.

## Results

During the median follow-up time of 178 (range, 48–1072) days, progression (arterial hypervascularization or > 1 cm diameter increase) was seen in 18 (16.9%) lesions in 12 (17.9%) patients (Supplementary Fig. 1). Progression rates at 3 and 6 months were 0.9% and 13.2%, and progression was encountered within 1 year in all lesions. Progression was diagnosed with arterial hypervascularization in 13 lesions, with > 1 cm increase in size in 3 lesions, and with both in 2 lesions. At the time of progression, 15 of 18 lesions were LI-RADS category LR-5 and three lesions were LR-4. The mean number of progressed lesions was 1.5 (range, 1–5) per patient. Out of 18 lesions progressed, 17 lesions were in patients who were receiving systemic treatment with sorafenib (either alone or combined with locoregional therapies).

Using ROC analysis, a cut-off value of 12.6 mm for lesion diameter (area under the curve, 0.749) was determined to have the highest sensitivity (55.5%) and specificity (67.0%) to predict progression during follow-up. While the progression rate was 25.6% in lesions > 12.6 mm, it was 11.9% in lesions ≤ 12.6 mm (*p* = 0.070).

Univariable analysis revealed that ECOG-PS 1 (*p* = 0.025), hypointensity at T1-weighted imaging (*p* = 0.028), hyperintensity at T2-weighted imaging (*p* < 0.001), and hyperintensity at DWI images (*p* = 0.007) were significant risk factors for progression during follow-up (Table 2). Additionally, underlying cirrhosis was associated with a higher risk of progression (20.9% vs. 4%, *p* = 0.065). The progression rates were 1/9 (11.1%) in lesions without sorafenib treatment and 17/97 (17.5%) with sorafenib treatment, and there was no correlation between sorafenib treatment (with or without SIRT) and progression (*p* > 0.99). There were also no significant differences between lesions with and without progression with respect to etiology of hepatitis B (*p* = 0.685), hepatitis C (*p* = 0.702) or alcoholic liver disease (*p* = 0.12), portal vein invasion (*p* = 0.288), Child-Pugh class (*p* > 0.99), BCLC stage (*p* = 0.296), extrahepatic metastasis (*p* = 0.534), alpha-fetoprotein (> 400 mg/dL, *p* = 0.434), diameter of index lesion (> 70 mm, *p* > 0.99), and presence of intralesional fat (*p* > 0.99).

**Table 2:** Univariable analysis of risk factors for progression

*N* = 106	No progression (*n* = 88)	Progression (*n* = 18)	Univariable analysis *p* value	Multivariable analysis *p* value
Size				
• > 10 mm	44 (50.0%)	8 (44.4)	0.667	
• ≤ 10 mm	44 (50.0%)	10 (55.6)		
Size				
• > 12.6 mm	29 (32.9)	10 (55.6)	**0.070**	0.148
• ≤ 12.6 mm	59 (67.1)	8 (44.4)		
Sorafenib				
• Yes	80 (90.9)	17 (94.4)	> 0.99	
• No	8 (9.1)	1 (5.6)		
Cirrhosis				
• Yes	64 (72.7)	17 (94.4)	**0.065**	**0.033**
• No	24 (27.3)	1 (5.6)		
Hepatitis B				
• Yes	10 (11.3)	1 (5.6)	0.685	
• No	78 (88.7)	17 (94.4)		
Hepatitis C				
• Yes	11 (12.5)	3 (16.6)	0.702	
• No	77 (87.5)	15 (83.4)		
Alcohol				
• Yes	39 (44.3)	12 (66.7)	0.12	
• No	49 (55.7)	6 (33.3		
ECOG-PS				
• 0	74 (84.1)	11 (61.1)	**0.025**	**0.030**
• 1	14 (15.9)	7 (38.9)		
Portal vein invasion				
• Yes	32 (36.3)	4 (22.2)	0.288	
• No	56 (63.7)	14 (77.8)		
Child-Pugh class				
• A	84 (95.4)	18 (100)	> 0.99	
• B	4 (4.6)	0 (0.0)		
BCLC				
• A	15 (17.0)	2 (11.1)	0.296	
• B	27 (30.6)	3 (16.6)		
• C	46 (52.4)	13 (72.3)		
Metastasis				
• Yes	18 (20.4)	5 (27.7)	0.534	
• No	70 (79.6)	13 (72.3)		
Alpha-fetoprotein				
• > 400	34 (38.6)	5 (27.7)	0.434	
• ≤ 400	54 (61.4)	13 (72.3)		
Index lesion				
• > 70 mm	14 (15.9)	2 (11.1)	> 0.99	
• ≤ 70 mm	74 (84.1)	16 (88.9)		
T1 intensity				
• Iso-high	34 (38.6)	2 (11.1)	**0.028**	0.996
• Low	54 (61.4)	16 (88.9)		
T2 intensity				
• High	36 (40.9)	17 (94.4)	**< 0.001**	**0.011**
• Iso-low	52 (59.1)	1 (5.6)		
Intralesional fat				
• Yes	14 (16.1)	3 (17.6)	> 0.99	
• No	73 (83.9)	14 (82.4)		
DWI (missing in 46 pts)				
• High	18 (36.7)	8 (88.9)	**0.007**	–
• Iso	31 (63.3)	1 (11.1)		

Bold type indicates *p* value < 0.1

Due to the considerable number of patients without DWI images at baseline (*n* = 46), signal intensity at DWI images was excluded from the multivariable analysis. Multivariable analysis identified underlying cirrhosis (*p* = 0.033), ECOG-PS (*p* = 0.030), and hyperintensity at T2 images (*p* = 0.011) as independent risk factors for progression.

The interreader agreement for the individual imaging features ranged from substantial to almost perfect (*k* = 0.66–0.84; Supplementary Table 2).

## Discussion

Our results show that underlying cirrhosis, higher ECOG-PS, and T2 hyperintensity are risk factors for the progression of non-hypervascular hepatobiliary phase hypointense lesions in patients with HCC. Additionally, although it was not incorporated into the multivariable analysis due to a high number of patients missing with DWI, diffusion restriction was significantly associated with progression during follow-up.

Diagnosis of HCC with imaging relies on arterial hypervascularity and venous wash-out, and hypovascular lesions detected on the hepatobiliary phase images are not considered in treatment stratification algorithms of HCC [9]. Recently, Renzulli et al proposed modern imaging criteria describing non-hypervascular hepatobiliary phase hypointense lesions without diffusion restriction as high-grade dysplastic nodules and showed 85.7% of the positive predictive value of diagnostic criteria [7]. However, HCC islets have been shown within high-grade dysplastic lesions on histopathological evaluation [14]. Similarly, centralized pathological review of patients with non-hypervascular hepatobiliary phase hypointense lesions has shown that only 8.1% of these lesions are low-grade dysplastic lesions or regenerative nodules, and the rest either progressed or early HCC or high-grade dysplastic nodules [6]. These findings indicate the need for additional parameters to differentiate progressed HCC lesions without arterial hypervascularization detected on hepatobiliary phase images.

Furthermore, these lesions are correlated with intrahepatic distal recurrence and shorter recurrence-free survival after curative therapies [10, 15]. T1 hypointensity, T2 hyperintensity, and diffusion restriction have been defined as risk factors for progressed HCC in non-hypervascular hepatobiliary phase hypointense lesions, which were also significant risk factors for progression in our cohort [6, 16]. Hyperintensity at T2 images was the only significant imaging parameter for progression in the multivariable analysis in our cohort. LI-RADS also incorporated T2 hyperintensity as an ancillary feature favoring malignancy [17]. Furthermore, according to modern imaging criteria proposed by Renzulli et al, non-hypervascular hepatobiliary phase hypointense lesions with diffusion restriction are classified as early HCC [7]. T2 hyperintensity correlates to small cell change, growth centers, nodule-in-nodule pattern, and higher grade [18, 19]. Additionally, due to iron and copper accumulation, regenerative or dysplastic nodules appear hypointense on T2 images [20].

In addition to hyperintensity at T2 images, cirrhosis and higher ECOG-PS score were associated with increased progression in our cohort. In patients with cirrhosis, sustained hepatic inflammation leads to genetic and epigenetic events that induce hepatocarcinogenesis. This process is mainly regulated by pro-inflammatory cytokines (like IL-6), which are shown to be associated with survival in patients with HCC [21–23], which are also correlated with higher ECOG-PS score [22]. Additionally, Iavarone et al have shown that higher ECOG-PS correlates with decreased radiological response rates in HCC patients receiving sorafenib treatment, which might have resulted from treatment interruption due to intolerance [24].

Our results suggest that these factors might lead to the identification of non-hypervascular HCC lesions, which should also be treated in patients allocated to locoregional therapies, especially when these lesions are not within the projected treatment area.

To the best of our knowledge, the existing studies on this topic investigated patients after local therapies or resection, or studied the natural course of non-hypervascular hepatobiliary phase hypointense lesions, and most of the studies evaluated Asian cohorts. Our cohort comprises HCC patients with a wide range of disease burdens (BCLC A-C), and the main underlying etiology was alcoholic liver disease (46.2%), which is representative of a Western cohort. Additionally, due to relatively advanced stages, median follow-up was shorter in our study, which also translated into a lower rate of progression than cohorts with chronic liver disease (16.2% vs. 28.2%) [11]. The majority of patients (61/67) were under systemic treatment for an existing HCC lesion in our study. Therefore, one might speculate that such a treatment could influence the hepatocarcinogenesis from precursor HCC lesions with high-grade dysplasia to overt HCC. However, this hypothesis was not supported by our cohort since a considerable number of non-hypervascular hepatobiliary phase hypointense lesions still progressed to HCC, and the univariable analysis with a *p* > 0.99 did not reveal any difference with regard to the factor “sorafenib.” Also, the effect of sorafenib is shown to be lower in hypovascular lesions [25].

Our study has some limitations. First, none of the lesions were histopathologically confirmed. However, radiological progression during follow-up provides secondary proof for pathological features. Second, some of these lesions theoretically received treatment with SIRT. But, due to lack of hypervascularity, we believe microsphere accumulation was rather the same as in the normal liver parenchyma and not in therapeutic doses. Also, in comparison to other patients, the progression rate was similar. And a major strength is that our study was a pre-planned subanalysis of patients randomized in the setting of prospective trial with blinded centralized image reading, and provides level B of evidence on this topic for the first time in literature.

In conclusion, our study shows that non-hypervascular hepatobiliary phase hypointense lesions detected in HCC patients are associated with a considerable rate of progression, and the presence of T2 hyperintensity, cirrhosis, higher ECOG-PS, and hyperintensity at DWI is associated with increased risk of progression. Therefore, during the decision-making process of locoregional therapies, such lesions with increased risk factors should be considered for further evaluation or treatment to improve the outcome of patients.

## Supplementary Information

ESM 1 (DOCX 243 kb)

## Abbreviations

BCLC: Barcelona Clinic Liver Cancer
DNA: Deoxyribonucleic acid
DWI: Diffusion-weighted imaging
ECOG-PS: Eastern Cooperative Oncology Group Performance Score
GRE: Gradient echo
LI-RADS: Liver Imaging Reporting and Data System
RFA: Radiofrequency ablation
ROC: Receiving operator curve

## Declarations

### Guarantor

The scientific guarantor of this publication is Max Seidensticker.

### Statistics and biometry

One of the authors (O.Ö.) has significant statistical expertise.

### Study subjects or cohorts overlap

The main results of the clinical trial have been previously reported in *Ricke J Hepatology 2019.*

### Methodology

• retrospective

• diagnostic or prognostic study

• multicenter study
