# Supplementary material for: Non-hypervascular hepatobiliary phase hypointense lesions detected in patients with hepatocellular carcinoma: a post hoc analysis of SORAMIC trial to identify risk factors for progression
Source: Eur Radiol. 2022 Jul 26;33(1):493–500. doi: 10.1007/s00330-022-09000-1 (PMC9755078; doi:10.1007/s00330-022-09000-1)

**Supplementary table 1. Reasons for exclusion**

| **Reason** | **Number of patients** |
| --- | --- |
| BCLC-D | 1 |
| Bilirubin > 1.5 mg/dL | 7 |
| Child-Pugh score > 7 | 10 |
| Lung metastasis | 4 |
| Other malignancies | 14 |
| Previous sorafenib treatment | 1 |
| Renal insufficiency | 4 |

**Supplementary table 2. Interreader agreement**

| **Imaging features** | **_K_ Value** |
| --- | --- |
| T1 intensity | 0.79 |
| T2 intensity | 0.75 |
| Intralesional fat | 0.84 |
| Intenstiy on DWI | 0.82 |
| Intenstiy on ADC | 0.66 |

Supplementary Figure 1


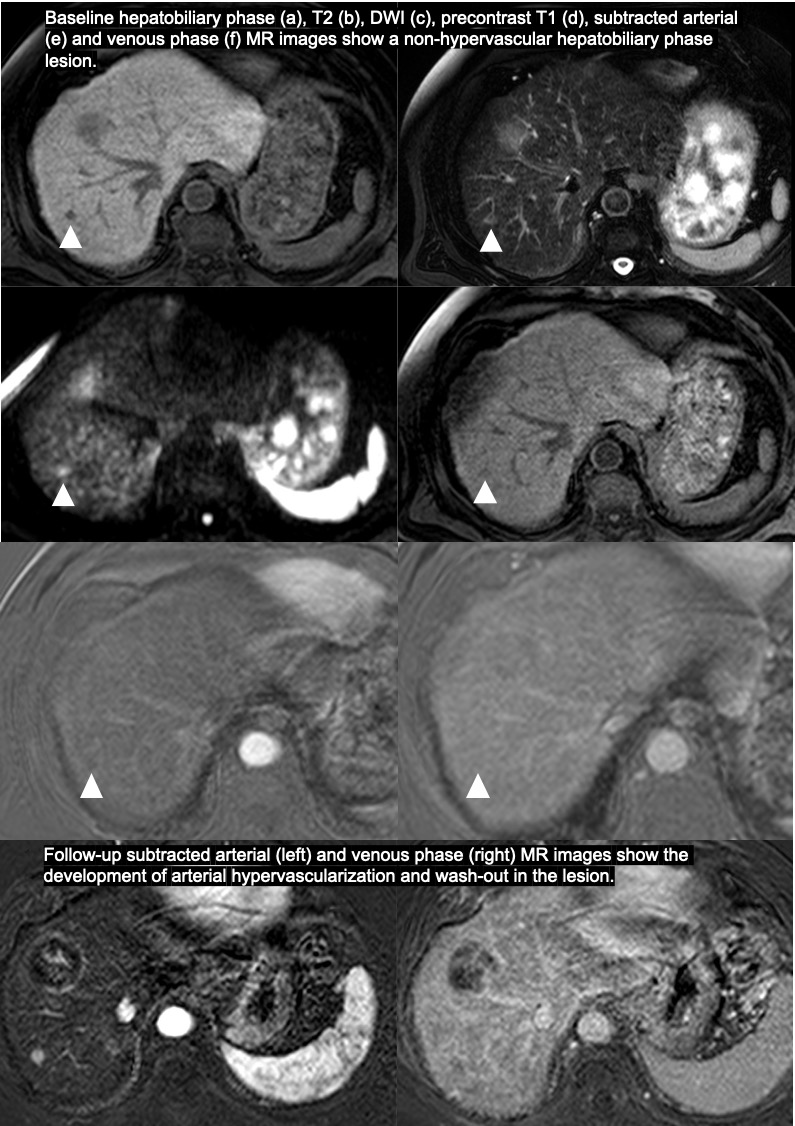

Supplement: Supplementary file 1 — (DOCX 243 kb) [file 330_2022_9000_MOESM1_ESM.docx]
